# Assessment of maternal knowledge on neonatal danger signs

**DOI:** 10.6026/9732063002001022

**Published:** 2024-09-30

**Authors:** Mahalakshmi N.B., Madhavi S., Rajendran K., Renuga R., Surya Surendran, Menaka J., Sivasubramanian N.

**Affiliations:** 1 KMCH College of Nursing, Coimbatore, Tamilnadu - 641048, India; 2 Department of Paediatric & Neonatology, KMCHIHSR, Coimbatore, Tamilnadu - 641048, India; 3 Nootan College of Nursing, Sankalchand Patel University, Visnagar, Gujarat - 384315, India

**Keywords:** Neonatal danger signs, maternal knowledge, healthcare-seeking behavior

## Abstract

Maternal knowledge of neonatal danger signs and subsequent healthcare-seeking behaviors are critical for early intervention and reducing neonatal mortality. This study aimed to assess these factors among mothers attending the Immunization Centre at KMCH Hospital, Coimbatore. A quantitative approach with a descriptive design was employed. Fifty mothers were selected via convenience sampling and administered a structured questionnaire. This tool encompassed socio-demographic variables, obstetric history, and a 30-item assessment on maternal knowledge of neonatal danger signs and healthcare-seeking behaviors. Findings revealed that while 80% of mothers exhibited moderate knowledge, only 16% demonstrated adequate knowledge of neonatal danger signs. Factors influencing healthcare-seeking behaviour included maternal education, family income and access to healthcare services. Comparative analysis with previous studies underscored consistent patterns of moderate knowledge but highlighted regional disparities in healthcare access and socio-economic impacts. The study emphasizes the critical need for targeted educational interventions to enhance maternal awareness of neonatal danger signs and promote early healthcare seeking. Improving maternal knowledge and behaviour could significantly mitigate neonatal morbidity and mortality rates in Coimbatore and similar settings. Future research should focus on sustained behaviour change and broader implementation of educational initiatives to improve maternal and neonatal health outcomes.

## Background:

The neonatal period, encompassing the first 28 days of life, is a critical time marked by high vulnerability to illness and death. Globally, neonatal mortality accounts for nearly 47% of all under-five child mortality, with approximately 2.4 million neonatal deaths reported in 2019 [1]. In India, the neonatal mortality rate (NMR) was 23 per 1,000 live births in 2018, highlighting the urgent need for improved neonatal care and early intervention strategies. Neonatal danger signs are vital clinical indicators of potentially life-threatening conditions requiring immediate medical attention [2]. These signs include symptoms such as stopped feeding, history of convulsions, fast breathing, severe chest in-drawing, no spontaneous movement, fever, low body temperature, jaundice, pus discharge from the umbilicus, cyanosis, vomiting and diarrhea [3]. Timely recognition and response to these signs are crucial for reducing neonatal morbidity and mortality[4]. Mother's health care seeking behavior plays a pivotal role in the timely management of neonatal illnesses. Understanding the level of knowledge and the determinants influencing this behavior is essential for designing effective interventions. Factors such as socio-economic status, educational level, cultural beliefs, and access to health services significantly impact a mother's ability to recognize and act upon these danger signs [5]. Research indicates that a lack of awareness and delays in seeking health care are major contributors to neonatal deaths [6]. In India, disparities in health care access and quality further exacerbate neonatal health outcomes. Rural areas often suffer from inadequate health infrastructure and limited availability of skilled health professionals, while urban centers face challenges related to overcrowding and unequal resource distribution. These disparities highlight the importance of targeted educational programs that address specific needs and barriers within different communities. Therefore, it is of interest to evaluate the level of knowledge and health care seeking behavior related to neonatal danger signs among mothers attending the Immunization Centre at KMCH Hospital.

## Methodology:

## Research design:

The study employed a quantitative research approach with a descriptive design [7, 8].

## Setting and population:

The study was conducted at the Immunization Centre of KMCH Hospital, a 1,000-bed super-speciality hospital in Coimbatore, India. The study population included mothers with newborns and infants aged between birth and one year who visited the center for immunization.

## Sample size and sampling technique:

A sample size of 50 mothers was selected for the study using a convenient sampling method.

## Data collection tool:

A structured questionnaire was developed, consisting of two sections: socio-demographic and obstetric characteristics and a 30-item knowledge assessment focused on health care seeking behavior for neonatal danger signs. The tool was validated by pediatric nursing experts and pilot-tested for reliability.

## Data collection procedure:

Formal permission for data collection was obtained from the chairman and the Head of the Pediatric Department at KMCH Hospital. The investigator introduced herself to the mothers and established rapport. The purpose of the study was explained and verbal consent was obtained from the mothers and their family members. Data were collected over two days using the structured knowledge questionnaire. Each participant was given approximately 15 minutes to complete the questionnaire.

## Data analysis:

The collected data were analyzed using both descriptive and inferential statistics. Descriptive statistics, such as frequencies and percentages, were used to summarize the demographic and obstetric characteristics of the participants (Table 1). Inferential statistics, including chi-square tests, were used to identify associations between socio-demographic variables and the mothers' knowledge of health care seeking behavior for neonatal danger signs (Table 2).

## Ethical considerations:

Ethical approval for the study was obtained from the institutional review board of KMCH Hospital. Informed consent was obtained from all participants, and confidentiality of the information provided was maintained throughout the study.

## Results:

**Figure 1:**
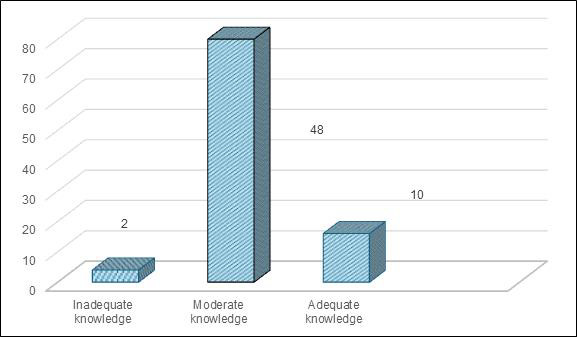
Bar graph showing distribution of sample as per their knowledge category

In the study of maternal and child health services (n=50), it was found that all participants (100%) had followed up with antenatal care (ANC). The frequency of ANC visits was higher in the group with four or more visits (54%) compared to those with fewer than four visits (46%) as shown in Figure 1. The majority of deliveries occurred in hospitals (56%), followed by health centers (40%), and only a small fraction at home (4%). Postnatal care (PNC) follow-up was very high, with 96% of participants adhering to it. Spousal accompaniment during ANC was noted in 90% of cases. Birth preparedness practices varied, with 50% saving money, 28% buying delivery materials, 12% arranging transportation, and 10% identifying a skilled birth attendant. None of the participants were unprepared for birth.

## Discussion:

In present study, we found that 80% (48) of mothers demonstrated moderate knowledge levels regarding neonatal danger signs, with only 16%(10) exhibiting adequate knowledge. This suggests a crucial gap in awareness that could impact early intervention and care-seeking behaviors for neonatal health issues. Strengthening educational initiatives targeting these specific danger signs is imperative to improve maternal responsiveness and ultimately neonatal health outcomes. Our findings align with studies conducted by Leta *et al.* (2022) and Ayele *et al.* (2022), highlighting similar patterns of moderate knowledge prevalence among mothers regarding newborn care [9, 10]. Our study's findings underscore the need for targeted educational campaigns focused on neonatal danger signs to bridge existing knowledge gaps and enhance maternal healthcare-seeking behaviors. This is consistent with Lassi ZS *et al.*'s (2019) findings, where educational interventions significantly improved maternal awareness and actions related to neonatal care. Furthermore, Emmanuel *et al.* (2021) demonstrated that socio-economic disparities can impact healthcare access, reinforcing the importance of inclusive healthcare policies to mitigate these barriers [11, 12]. The level of knowledge and health care seeking behavior regarding neonatal danger signs among mothers varies significantly across different regions and is influenced by several factors. Studies indicate that knowledge of neonatal danger signs is generally low, with only 34.3% of mothers in southwest Ethiopia and 45.78% in Gondar, Ethiopia, demonstrating good knowledge[13]. Factors such as maternal age, education, occupation, and antenatal care (ANC) attendance are significantly associated with better knowledge and health-seeking behavior. For instance, mothers aged 35-49, those with primary education, and those who received information on neonatal danger signs during ANC visits were more likely to have good knowledge [14, 15] In conclusion, while our study aligns with previous literature in confirming the influence of education and culture on maternal knowledge of neonatal danger signs, it enriches the understanding by highlighting the dual influences of occupation and income on healthcare-seeking behaviors. These comparative insights underscore the complexity of socio-demographic factors in shaping maternal healthcare decisions and advocate for tailored interventions that address both educational and economic disparities to improve maternal and neonatal health outcomes effectively.

## Figures and Tables

**Table 1:** Socio-demographic characteristics of mothers (n=50)

**S. No**	**Demographic Variable**	**No. of Samples**	**Percentage (%)**
1	Age in years		
	≤20	3	6
	21-25	40	80
	26-30	7	14
	≥31	0	0
2	Education Status of Mother		
	Illiterate	0	0
	Primary	3	6
	Secondary	2	4
	Graduate	37	74
	Post Graduate	8	16
3	Education Status of Father		
	Illiterate	0	0
	Primary	4	8
	Secondary	4	8
	Graduate	25	50
	Post Graduate	17	34
4	Occupation of Mother		
	Homemaker	25	50
	Self-employed	13	26
	Government	5	10
	Private	7	14
5	Occupation of Father		
	Government	10	20
	Private	22	44
	Business	10	20
	Farmer	8	16
6	Religion		
	Hindu	36	72
	Christian	10	20
	Muslim	4	8
	Others	0	0
7	Family Income		
	<5000	28	56
	5000-10000	13	26
	10000-20000	6	12
	>20000	3	6
8	Area of Residence		
	Urban	36	72
	Rural	14	28
9	Type of Family		
	Nuclear Family	30	60
	Joint Family	20	40
10	Place of Previous Delivery		
	Hospital	48	96
	Home	2	4
11	Source of Information		
	Family Members	10	20
	Friends	5	10
	Relatives	5	10
	Mass Media	12	24
	Health Workers	18	36
	Others	0	0
12	Presence of Medical Personnel in Family		
	Yes	28	56
	No	22	44

**Table 2:** Obstetric characteristics of mothers (n=50)

**S. No**	**Obstetric Characteristics**	**No. of Samples**	**Percentage (%)**
1	Age at First Pregnancy		
	<20	15	30
	20-29	30	60
	≥30	5	10
2	Number of Deliveries		
	1	17	34
	2	25	50
	3	8	16
3	History of Abortion		
	1	5	10
	02-Apr	0	0
	≥5	0	0
	No	45	90
4	History of Stillbirth		
	Yes	2	4
	No	48	96
5	Total Number of Children		
	1	18	36
	2	28	56
	3	4	8
